# Genetic transformation to integrate two expression cassettes into the genome of yeast Pichia pastoris

**DOI:** 10.1186/1753-6561-8-S4-P254

**Published:** 2014-10-01

**Authors:** João Victor Verçosa, Edson Junior Carmo, Spartaco Astolfi Filho

**Affiliations:** 1 Universidade Federal do Amazonas (UFAM), Instituto de Ciências Biológicas (ICB), Manaus, AM, Brazil

## Background

*Pichia pastoris *is a methylotrophic species of yeast, which means that it can grow on methanol as its sole carbon and energy source [1]. The *Pichia *expression system has several advantages: short length of the oligosaccharide chains added to proteins, correct folding and very high cell densities [2]. *P. pastoris *vectors are designed for homologous integration into either AOX I locus, one of the two homologous AOX I genes present in this species or his4 locus. Gene insertion events at the AOX I (GS115) loci arise from a single crossover events between the loci and any of the three AOX I regions in the vector, the AOX I promoter, the AOX I transcription termination region (TT), or sequences even further downstream of AOX I (3′ AOX I) [3]. In either GS115 gene insertion events at his4 locus arise from a single crossover event between his4 locus in the chromosome and His4 gene of the vector. This results in the insertion of one or more copies of the vector in his4 locus [3].

## Methods

Recombinants clones *P pastoris *GS115 (phenotype His^+^/Mut^s^) with pPG Vector (pPIC9/ glucoamylase cDNA of *Aspegillus awamori*) integrad in the AOX locus were transformad with pPAmy vector (pPIC9 vector/α-amylase gene of *Bacillus licheniformis*), both previouly contructed in our laboratory. The pPAmy was linearizad by digesting with *Sac*I enzyme to HIS4 locus integration. Transformation was carried out by electroporation of freshly prepared competent cells. The electroporated cells were recoved in 1 M sorbitol and spread onto MM agar plates containg starch 1 % (p/v) to identify positive clones. Transformants with larger halos and clear were considered as positive recombinant strains expressing tow enzymes in comparition the control strain (Strain previously constructed). Positive strains were then inoculated in 50 ml BMGY at 28 C for 48 h in shaking flasks. The cells were harvested by centrifugation and then grown in 25-ml BMMY with methanol induction (0,5% [v/v]) at 28 C for 120 h. The supernatants were collected by centrifugation and subjected to enzyme activity assay by Fuwa and DNS methods. Extracellular culture supernatant samples were used to proteins detection by SDS-PAGE 10%.

## Results and conclusions

A total of 744 transformants were screened on MM plates. Eight positive transformants with the highest halos amylase activities were analyzed by SDS-PAGE. The gel electrophoresis revealed expression two protein. One protein band with molecular weight of 116 kDa and another with 58 kDa, correponding to glucoamylase and α-amylase respectively. When we compared the protein pattern of secreted enzymes is agree with protein pattern observed in control supernadant, previously studed in our laboratory. However, in preliminary analyse, none enzymatic activety was detectad in enzymatc assay by Fuwa and DNS methods. However, new transformations and integrations strategies has been performing by study group.
